# Creativity in Generative Musical Networks: Evidence From Two Case Studies

**DOI:** 10.3389/frobt.2021.680586

**Published:** 2021-08-02

**Authors:** Rodrigo F. Cádiz, Agustín Macaya, Manuel Cartagena, Denis Parra

**Affiliations:** ^1^ Department of Electrical Engineering, Faculty of Engineering, Pontificia Universidad Católica de Chile, Santiago, Chile; ^2^ Music Institute, Faculty of Arts, Pontificia Universidad Católica de Chile, Santiago, Chile; ^3^ Department of Computer Science, Faculty of Engineering, Pontificia Universidad Católica de Chile, Santiago, Chile

**Keywords:** generative models, music, deep learning - artificial neural network (DL-ANN), VAE (variational AutoEncoder), GAN (generative adversarial network), creativity

## Abstract

Deep learning, one of the fastest-growing branches of artificial intelligence, has become one of the most relevant research and development areas of the last years, especially since 2012, when a neural network surpassed the most advanced image classification techniques of the time. This spectacular development has not been alien to the world of the arts, as recent advances in generative networks have made possible the artificial creation of high-quality content such as images, movies or music. We believe that these novel generative models propose a great challenge to our current understanding of computational creativity. If a robot can now create music that an expert cannot distinguish from music composed by a human, or create novel musical entities that were not known at training time, or exhibit conceptual leaps, does it mean that the machine is then creative? We believe that the emergence of these generative models clearly signals that much more research needs to be done in this area. We would like to contribute to this debate with two case studies of our own: TimbreNet, a variational auto-encoder network trained to generate audio-based musical chords, and StyleGAN Pianorolls, a generative adversarial network capable of creating short musical excerpts, despite the fact that it was trained with images and not musical data. We discuss and assess these generative models in terms of their creativity and we show that they are in practice capable of learning musical concepts that are not obvious based on the training data, and we hypothesize that these deep models, based on our current understanding of creativity in robots and machines, can be considered, in fact, creative.

## 1 Introduction

The field of deep learning (DL), one of the branches of artificial intelligence (AI), has become one of the most relevant and fast-growing research and development areas of recent times, especially since 2012, when an artificial neural network (ANN) called AlexNet (Krizhevsky et al., 2012) surpassed the most advanced image classification techniques to the date (Briot et al., 2020). This AI boom has happened because of three factors: first, today there is much more data available, second, there are much faster and more powerful computers available to researchers and third, technical advances. In particular, breakthroughs in the theory of ANNs, such as new training methods, convolutional networks, recurrent networks with short and long term memory, regularization techniques such as dropout, generative and transformer models, among others. These advances have allowed for the design and implementation of very sophisticated and complex AI models.

Indeed, DL models have been proven useful even in very difficult computational tasks, such as solving very difficult inverse problems with great precision (Goodfellow et al., 2016, 12). These approaches have the advantage that all parameters are objectively computed at the training stage, minimizing the error between predictions and the results provided by the training data. Training processes tend to be of high computational load, but once the training is finished, ANN-based reconstructions are extremely fast. However, classification and regression are perhaps not the most impressive applications of DL. There is increasing evidence showing that DL models can also generate very realistic audiovisual content, apparently at the same level of expert humans. In particular, variational auto-encoders (VAEs) and generative adversarial networks (GANs) are the most widely used generative strategies, yielding very interesting results, especially in the form of deep-fakes or deep video portraits (Kim et al., 2018).

Research in the field of robot musicianship has a rich history (Rowe, 2004) and it has experienced an increasing interest in recent times (Bretan and Weinberg, 2016). Currently there are robotic performers that can achieve very expressive performance levels, particularly with reinforcement learning approaches (Hantrakul et al., 2018) and machines that can compose music in real-time based on inference rules (Cádiz, 2020), or with direct interaction with its environment and people (Miranda and Tikhanoff, 2005). However, the question of creativity in robot musicianship remains elusive. We would like to contribute to the creation of better robotics composers or improvisers by studying the creativity of DL generative musical networks and identifying musical elements that could enlighten the discussion.

In this article, we study the use of generative models for musical content creation by means of a literature survey as well as by presenting two case studies and examining them under the light of computational creativity theory. The first use case describes the implementation and usage of a VAE model to encode and generate piano chords directly in audio, which we call TimbreNet. The second use case is a generator of musical piano rolls based on the StyleGAN 2 network architecture. Piano rolls are a widely used two-dimensional representation of musical data, very similar to a musical score in the sense that the *x*-axis represents time while pitches are encoded in the *y*-axis. We believe that both generative models, even though they have different architectures and music representations, exhibit behavior that could be classified as creative, as they can represent musical concepts that are not obvious based on the training data, and also exhibit conceptual leaps.

This article is structured as follows. In section 2, we discuss the most important generative models and show how they are able to create content. In section 3, we introduce the concept of computational creativity and provide a state-of-the-art review on the topic, including the most used ways for assessing creativity in computational systems. In section 4, we provide two case studies of generative networks that we think exhibit creative behavior. In section 5, we describe a simple perceptual survey we created to subjectively assess traits of creativity of the results of one of our models. In section 6, we discuss these case studies under the light of computational creativity theory and assess their creativity. Finally, in section 7, we present our main findings and layout ideas for future work.

## 2 Deep Generative Models

According to Goodfellow et al. (2014), DL promises that we can build models that represent rich and hierarchical probability data distributions, such as natural images or audio, with great accuracy. This potential of DL makes perfect sense for music, being in essence very rich, structured, and also hierarchical information encoded in either a two-dimensional format (a score or a piano roll) or as one-dimensional array of audio samples. It is no surprise then that this amazing growth of DL in recent years has also greatly impacted the world of music and of machine musicianship.

As we stated before, perhaps one of the most interesting aspects that these networks can do now, apart from classification and regression, is the generation of content. In particular, ingenious network architectures have been designed for the generation of images, text, paintings, drawings or music (Briot et al., 2020). In the music realm, perhaps one of the most relevant research devoted to music generation is being carried out by the Magenta project.^1^, a part of Google Brain. The goals of Magenta is not only to automatically generate new content, but to explore the role of ML as a tool in the artistic and creative process.

One of the most important aspects of generative DL approaches for music is their generality. As Briot et al. (2020) emphasize: “As opposed to handcrafted models, such as grammar-based or rule-based music generation systems, a machine learning-based generation system can be agnostic, as it learns a model from an arbitrary corpus of music. As a result, the same system may be used for various musical genres. Therefore, as more large-scale musical datasets are made available, a machine learning-based generation system will be able to automatically learn a musical style from a corpus and to generate new musical content”. Contrary to rule-based structured representations, DL is very appropriate for handling raw unstructured data, and to extract higher-level information from it. We believe that this particular capacity makes DL a suitable technique for novel musical content generation.

Almost exclusively, these efforts aimed towards musical content creation are based on generative models, which are unsupervised models that intend to represent probability distributions over multiple variables (Goodfellow et al., 2016, 645). Some approaches estimate a probability distribution function explicitly, while others support operations that require some knowledge of it, such as drawing samples from the distributions. Although several models can generate content, there are two that are the most promising and relevant today: VAEs and GANs (Charniak, 2018, 137).

VAEs are a probabilistic type of ANNs known as auto-encoders, which are functions whose output is nearly identical to the input (Charniak, 2018, 137). They are encoders because to generate the output, the network must have learned to represent the input data in a much more compact way, more specifically a low-dimensional space, known as a latent space. In a VAE, samples are drawn from the latent space to generate new outputs. As it is not possible to fill an entire latent space with only training data, some points in this space will inevitably generate outputs that were previously unknown to the network, an apparent sign of creativity.

More specifically, the loss function of a VAE (Kingma and Welling, 2014) can be described by the equation:

$${ℒ}_{\text{VAE}}=E_{q\left(z|x\right)}\left[\text{log}p\left(x|z\right)\right]-\text{KL}\left(q\left(z|x\right)∥p\left(z\right)\right)$$ (1)

The first term in Eq. 1 corresponds to the reconstruction loss, which is the expectation over the log-likelihood of the reconstructed data points using the decoder p(x|z), where *z* is sampled from the encoder q(z|x). The second part of the equation is considered a regularization term, the Kullback-Leibler (KL) divergence between the encoder distribution q(z|x) and p(z). The prior distribution is placed over the encoder and decoder parameters and in this article we use a Gaussian prior with mean zero and variance one, since it facilitates the generation of new samples from the latent space, it has an analytical evaluation of the KL divergence in the loss function, and the non-linear decoder can mimic arbitrarily complicated distributions if necessary starting from the prior Gaussian distribution. This loss function of VAEs decreases as the input and output data are alike, and in every iteration, a VAE network learns to represent the input space in a more efficient and compressed form. The decoder part of the network can thus generate novel output that share a lot of the characteristics of the input space.

Another promising research in DL is related to the development of Generative Adversarial Networks (GANs), which represent a significant shift from traditional DL architectures. In GANs, two ANN work against each other in adversarial training to produce generative models (Kalin, 2018, 9). More formally, GANs (Goodfellow et al., 2014) have provided a new framework for estimating generative models via what is called an adversarial process, in which two models are simultaneously trained. In this approach, the input data distribution is estimated by a generative model (G), while a discriminator model (D) evaluates the probability that a freshly generated output provenances is indeed from the training data rather than from the generator G. The whole idea of this approach is to make the generative model G so good that eventually D might be fooled by a false input. If this happens, it means that G is generating fake data that is indistinguishable from real data, also a possible indication of creativity.

This process can be summarized in Eq. 2, where the goal with the adversarial training is to find the function *D* which maximizes the log probability of correct cases, while the generator *G* minimizes the log-probability of the discriminator being correct.

$$\underset{G}{\text{min}}\underset{D}{\text{max}}V\left(D,G\right)=E_{x∼p_{\mathrm{data}}\left(x\right)}\left[\text{log}D\left(x\right)\right]+E_{z∼p_{z}\left(z\right)}\left[\text{log}\left(1-D\left(G\left(z\right)\right)\right)\right]$$ (2)

Once trained, these networks can convert random noise into highly realistic content, such as images or audio signals. There are several advantages of this approach: GANs generalize well with limited data and they can conceive new scenes from small datasets, but perhaps, the most important aspect is that they make simulated data look highly realistic (Kalin, 2018, 10).

### 2.1 Musical Generative Networks

In the musical field, generative models such as the ones we previously discussed have been gaining popularity in recent times for the creation of audible content. We now provide a literature review of the most relevant works for music creation based on these two architectures.

Hadjeres et al. (2016) created DeepBach, a neural network capable of modeling polyphonic music and pieces in the anthem genre, which harmonizes Bach-style choral in a very convincing way. Oord et al. (2016) created Wavenet, a network that renders audio files at the sample level. Wavenet has been shown to produce good results in human voice and speech. Engel et al. (2017), using NSynth, a very large dataset of sound for digital synthesis, were able to improve both the qualitative and quantitative performance of WaveNet. Their model learns a manifold of embeddings that allows for instrument morphing, a meaningful way for interpolating timbre that results in new types of realistic and expressive sounds. Sturm et al. (2016) have used generative models for music transcription problems. They specifically designed generative long short-term memory (LSTM) models, for the task of music transcription and composition. Roberts et al. (2018) created MusicVAE, a network designed for the generation of compact latent spaces that can be later interpolated for the generation of content. Yang et al. (2017) created MidiNet, a convolutional adversary generation network able to produce melodies in the MIDI format. Dong et al. (2018a) created MuseGAN, an adversarial network for symbolic music and accompaniment, in this case in the rock genre. Roberts et al. (2017) designed a VAE for the generation of a variety of musical sequences at various bar scales: 2-bar, 16-bar or 32-bars. Yamshchikov and Tikhonov (2020) propose a novel DL architecture labeled as Variational Recurrent Autoencoder (VRASH), that used previous outputs as additional inputs, forming a history of the analyzed events. VRASH “listens” to the notes already output and uses them as a feed for “historic” input. This is the first application of such a generative approach to the generation of music rather than text. Weber et al. (2019) were able to generate novel melodies via a ANN model that ensures, with high probability, consistency of melody and rhythm with a target set of sample songs. A unique aspect of this work is that they propose the usage of Perlin noise in opposition to the more widely used white noise in the context of VAEs.

In the field of audio processing, impressive advances have been made in the last two years. As an example, we can cite Spleeter (Hennequin et al., 2019), a music source separation tool for up to five simultaneous voices based on deep learning. This task is extremely hard when tackled with traditional signal processing approaches. Another interesting example is the Differentiable Digital Signal Processing (DDSP) library (Engel et al., 2020), created by Magenta, which enables direct integration of classic signal processing elements with the power of deep learning. This approach achieves high-fidelity audio generation without the need for large models or adversarial architectures. DDSP models are similar to vocoder systems, which are physically and perceptually motivated, and directly generate audio with oscillators, and do not work by predicting waveforms or Fourier coefficients, as traditional methods do.

In section 4 we will elaborate on two generative models that we have built aimed towards the generation of audio-based musical chords and symbolic piano roll-based short musical sequences. In the specific case of chords generation, a significant amount of research is aimed towards chord recognition (Humphrey et al., 2012; Zhou and Lerch, 2015; Deng and Kwok, 2016; Korzeniowski and Widmer, 2016), in detriment of chord generation. The first study case what we present below is based on GanSynth (Engel et al., 2017), a GAN model that when its latent vector is sampled, it generates a complete audio excerpt, allowing for a smooth control of features such as pitch and timbre. Our model is based on GanSynth, but it was tuned for the specific case of chord sequences. In terms of piano roll sequence generation, MuseGAN (Dong et al., 2018a) is probably the most well-know model targeted for this specific musical format. Our second case study uses piano rolls instead of images in a network previously trained with only real-world images.

## 3 Computational Creativity

A very important question in the field of artificial intelligence is whether computers or robots can be creative. This is a very difficult research topic, as scientists have only embraced the study of human creativity in recent times (Sawyer, 2006, 3). According to Brown (1989), four distinct approaches have dominated the study of creativity: 1) an aspect of intelligence; 2) a largely unconscious process; 3) an aspect of problem-solving; and 4) an associative process. Nowadays, the study of creativity in humans has settled into what is called the socio-cultural approach, an interdisciplinary effort to explain how people are creative and their social and cultural contexts (Sawyer, 2006, 4).

It is a consensus that creativity can be defined as “the ability to generate novel, and valuable, ideas” (Boden, 2009). This definition implies the generation of “something that is both original and worthwhile” (Sternberg and Sternberg, 2012), or a “conceptual leap” by the combination of existing knowledge (Guzdial and Riedl, 2019). These “ideas” or “somethings” can take the form of intangibles, such as a scientific theory, a mathematical theorem, a musical composition, a neural network, a poem, or a joke; or even tangible physical objects, such as an invention, a robot, a mechanical tool, a chemical, a printed literary work, a sculpture, a digital circuit, or a painting. The notion of novelty is crucial for this understanding of creativity. But in addition, as previously stated, Boden (2009) emphasizes that creativity should be “valuable”. This implies a subject-dependent evaluation, as what influences the assessment we make of something is not only its features or objective properties, but rather how such a thing is produced and presented (Moruzzi, 2018). It is also worth emphasizing that novelty often implies unpredictability and uncertainty, especially in the case of musical creativity (Daikoku et al., 2021).

Carnovalini and Rodà (2020) observe that “the usual experience with machines is that we humans give a set of instructions to the machine along with some initial data (the input), and we expect the machine to behave in a way that is fully deterministic, always giving the same output when the same input is given”. This idea of deterministic robots is apparently very opposed to the whole notion of creativity, which supposes something novel and valuable. This notion of “novelty” is understood by Grace and Maher (2019) as “violated-expectations” models. However, as Mumford and Ventura (2015) point out, a “common misconception among non-specialists is that a computer program can only perform tasks which the programmer knows how to perform (albeit much faster). This leads to a belief that if an artificial system exhibits creative behavior, it only does so because it is leveraging the programmer’s creativity”.

There are other ways of conceptualizing creativity. In particular, the categories of combinatorial, exploratory and transformational creativity, proposed by Boden (2004), are very enlightening. The first one is about making unfamiliar combinations of known ideas. The second one involves a structured conceptual space that is explored. The third category implies changing this conceptual space allowing new ideas to become possible. All of these categories are related to the conceptual leaps proposed by Sternberg and Sternberg (2012) in different degrees.

Another important aspect of creativity is the ability to autonomously evaluate outcomes, to “know when to stop” (Moruzzi, 2021). This aspect of creativity is crucial to determine whether the produced outputs work or not and reminds us that the process of creativity requires hard work, that it does not happen by pure magic. This autonomy means that the creative agent should be the one performing the assessment, without external influence.

Creativity is usually attributed to humans. However, as Park (2019) asks: When we regard something as artwork, should it be exclusively created, selected, and combined by human beings? We are used to the idea that humans can create things or ideas that other humans judge to be “new”–this happens almost every day in every domain. But computers can also produce outputs that can be thought of being new. For example, Cope (1996) developed computer algorithms which he labeled as “Experiments in Musical Intelligence (EMI)”, that allowed computers to generate novel compositions in a particular musical style, two decades before the rise of deep learning techniques. It is no surprise, then, that the study of the phenomenon of creativity has been extended to computers and machines, under the label “Computational creativity”, which is a field of inquiry seeking the modeling, simulation, or replication of creativity inside a computer. This field is interdisciplinary by nature, with links to traditional fields such as artificial intelligence, psychology, the arts, or philosophy. It is also known as creative computation, creative computing, or artificial creativity.

The goals of computational creativity are not only to design and build computer systems capable of achieving or enhancing human-level creativity, but also to better understand how human creativity works. In the particular case of deep generative networks, one of the most interesting and current theoretical research trend is to determine if these generative networks are creative or not and to what extent. Karimi et al. (2018) define creative systems as those intelligent systems that are capable of performing creative tasks in isolation or collaboration with other systems. These systems are creative because their results are judged as such by their human counterparts (Colton et al., 2015; Elgammal et al., 2017). There even exist Turing-style tests to assess creativity from machines that create artworks, by asking machines to create art that is indistinguishable from human-created works.

The question of how can machines and robots be creative is far from settled. On the one hand, there are authors, such as Hertzmann (2018), who argue that the current AI technology is not yet able to create since to do it requires “intention, inspiration, and desire to express something”. However, the advances in AI open for music, as did photography with paintings more than 100 years ago, the possibility of generating new forms of artistic creation. It is possible to understand AI as a technology that can increase and enhance human capabilities (Carter and Nielsen, 2017). On the other hand, authors such as Elgammal et al. (2017) have no problem in considering their systems creative. As evidence, they have created an architecture of ANNs labelled CAN (Creative Adversarial Networks), which can look at visual art and learn the artistic style inherent in the works with which they were trained. Then, by modifying certain parameters of the network, the authors argue that they become creative because they are capable of generating new art that deviates from the styles that were previously learned. Similarly, Guzdial and Riedl (2019) present a novel training method for neural networks called Combinets, a more general approach for reusing existing trained models to derive new ones without retraining via recombination. In a sense, they can make a DL network “creative”, in the sense that it is able to represent new knowledge as a combination of particular knowledge from previous existing cases. Another important evidence towards the existence of creativity in machines is presented by Wyse (2019), who examined five distinct features typically associated with creativity, and provided examples of mechanisms from generative DL architectures that give rise to each of these characteristics, producing very strong evidence in favor of DL architectures being creative.

Another unresolved topic in the computational creativity field is the evaluation of generative systems in terms of their creativity (Ritchie, 2019). As Moruzzi (2018) illustrates: “The subjective judgments and biases which come with the evaluation of something as creative make it impossible to objectively answer the question “Can a computer be creative?” What we are measuring when we provide an answer to this question, in fact, are not the computer’s accomplishments but instead our subjective evaluation of them. We can then try to analyze not just the creativity exhibited by the outcome produced by the computer but, instead, the intention of the computer in producing it. In other words, we can judge whether the computer produced its outcome intentionally, i.e., consciously intending to produce exactly that outcome. We should then rephrase the question and ask: “Can a computer be intentionally creative?””.

We have identified four strategies for the evaluation of creativity in robots and algorithms reported in the literature. The first one follows what Jordanous (2019) calls a “creative-practitioner-type approach, producing a system and then presenting it to others, whose critical reaction determines its worth as a creative entity”, even in real-time (Collins, 2007). The second one is described by Carnovalini and Rodà (2020): “have the author of the system describe the way it works and how it can be considered creative or not, and to what degree.” A third one is to evaluate artificially generated music in a concert setting, just as normal auditors would assess a live musical situation (Eigenfeldt et al., 2012; Sturm et al., 2018), or in a museum-like setting for the case of the visual arts (Edmonds et al., 2009). Finally, a fourth approach that we can identify is described in (Yang and Lerch, 2020), who propose “informed objective metrics” to complement a subjective evaluation by a human. For example, some metrics can determine how well a computer-generated music can “fit” a particular musical genre.

In the following section we will describe two case studies and discuss their creativity under the light of what we have presented in this section. In particular, we will focus on the aspects of novelty, in the sense that the models produce something that is not expected, value, by assessing whether novel outputs make sense and function well in their context, and conceptual leaps, understood as the reuse of a particular type of knowledge to produce a different kind. For the purposes of this article, we will be using the second evaluation strategy, as we are the authors of both models.

## 4 Case Studies

We now present two case studies: TimbreNet, an ANN based on the architecture of GanSynth (Engel et al., 2017) that can generate novel chords directly in audio format and StyleGAN Pianorolls, a generative model, based on StyleGAN 2 (Karras et al., 2020b), that can create novel musical excerpts in the form of piano rolls.

### 4.1 TimbreNet: A Creative Chord Generator

**FIGURE 1:**
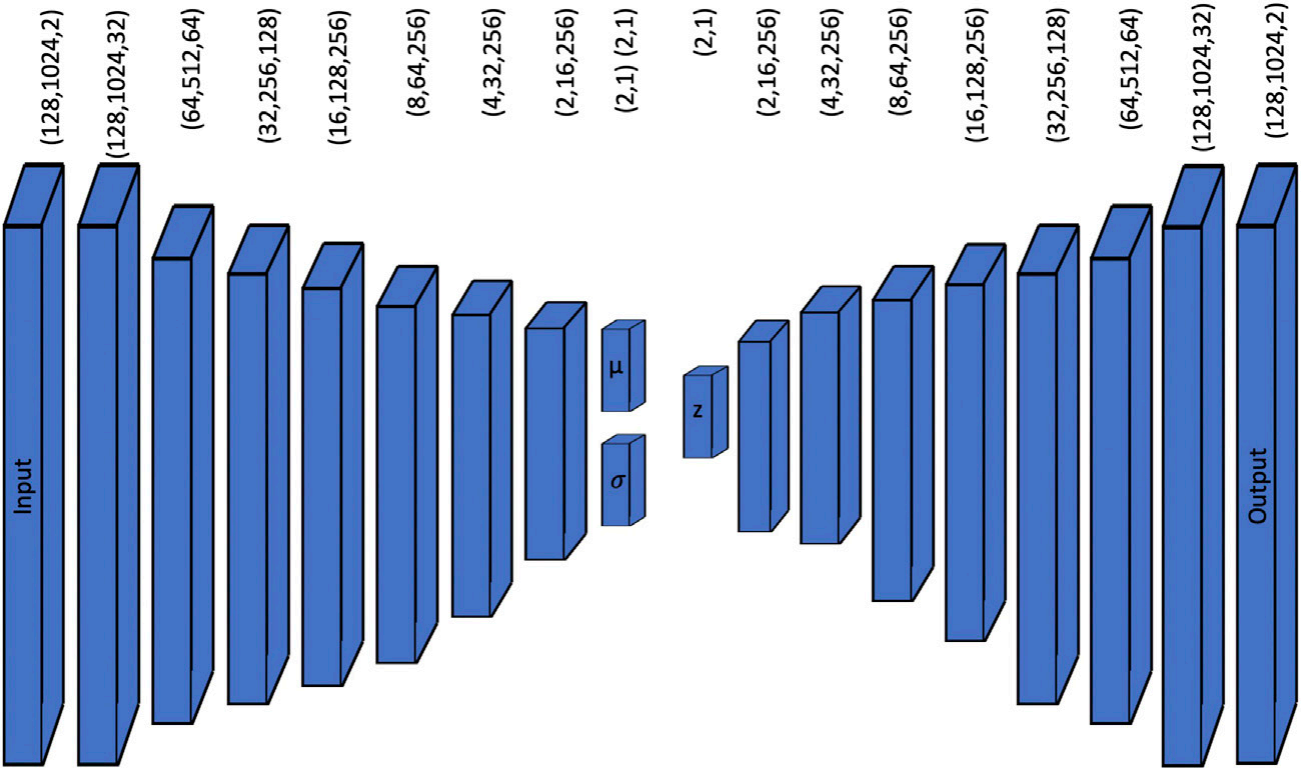
Architecture of our VAE model for chord synthesis for the case *L* = 2. The encoder takes a (128,1024,2) MFCC image and passes it through several downsampling layers until it compacts the data into a low-dimension latent space *z*. The decoding process samples the latent vector using a Gaussian distribution of mean *μ* and standard deviation *σ*, and passes it through several upsampling layers until a (128,1024,2) output is obtained that is later converted to an audio signal.

The network architecture is presented in Figure 1. Our design goal was to generate a useful tool for musical composition, by means of the latent space exploration. A VAE-based model can accept inputs directly from the user in contrast to GAN-based models where the input is random noise. Although it is possible to mimic this behavior with conditional GANs, we opted for a VAE to obtain an explicit latent space representation of the input data. We based the encoder architecture on the discriminator structure of GanSynth (Engel et al., 2017) and the decoder architecture from its generator.

The encoder takes a MFCC (Mel Frequency Cepstral Coefficients) image of dimensions (128,1024,2) and passes it through one two-dimensional convolution layer with several additional filters generating a (128,1024,32) output that is fed to a series of 2 two-dimensional convolution layers with the same size padding and a Leaky ReLU non-linear activation function in cascade with 2 × 2 downsampling layers. This process keeps halving the images’ size and duplicating the number of channels until a (2,16,256) layer is obtained. Then, a fully connected layer outputs a (2L,1) vector, the latent space, that contains *L* means and *L* standard deviations for posterior sampling. We trained models with different sizes for *L* (specifically 3, 4, 8, 16, and 32), which is a meta-parameter that determines the dimension of the latent space. Figure 1 displays the network structure for the case *L* = 2.

The sampling process begins with a (*L*,1) mean vector and a (*L*,*L*) standard deviation diagonal matrix that is used for sampling the latent vector *z* from a normal distribution with mean *μ* and standard deviation *σ*. The *z* latent vector is fed to the decoder in cascade with a fully connected layer that generates a (2,16,256) output that then is followed by a series of two transposed convolutional layers in series with an 2 × 2 upsampling layer that keeps doubling the size of the image and halving the number of channels until a (128,1024,32) output is achieved. This output passes through a final convolutional layer that outputs the (128,1024,2) MFCC spectral representation of the generated audio. This spectral representation can be converted into an audio excerpt by inverse MFCC and STFT transformations.

#### 4.1.1 Dataset and Model Training

Our dataset consisted on 43,200 recordings of tertian triads played at different keys, dynamic levels and octaves, performed by the second author on a piano. A triad is a chord containing three notes and a tertian chord is constructed by adding up notes separated by a major or minor third. Each recording was done in Ableton Live with a duration of 4 s, and a 16 kHz sampling rate. Piano keys were pressed for 3 s and then released during the last second. This dataset format has the same structure as the one used in Engel et al. (2017).

The base notes of the chords were the twelve notes of the western musical scale across three octaves giving a total of thirty-six base notes. For each base note, we recorded four different types of triads (major, minor, augmented, and diminished). We also recorded chords at three different levels of dynamics: f (forte), mf (mesoforte) and p (piano). For each combination, we produced ten different recordings for data augmentation purposes, as each recording is not an exact repetition of any other one, producing a total of 4,320 data examples and then we used data augmentation techniques to have a total of 43,200 examples. This dataset can be downloaded from the github repository of the project.^2^.

We decided to use an MFCC representation of the audio samples for the input and output data, a design decision that has been proven to be very effective when working with convolutional networks designed for audio content generation (Engel et al., 2017). Magnitude and unwrapped phase appear codified in different channels of the image.

**FIGURE 2:**
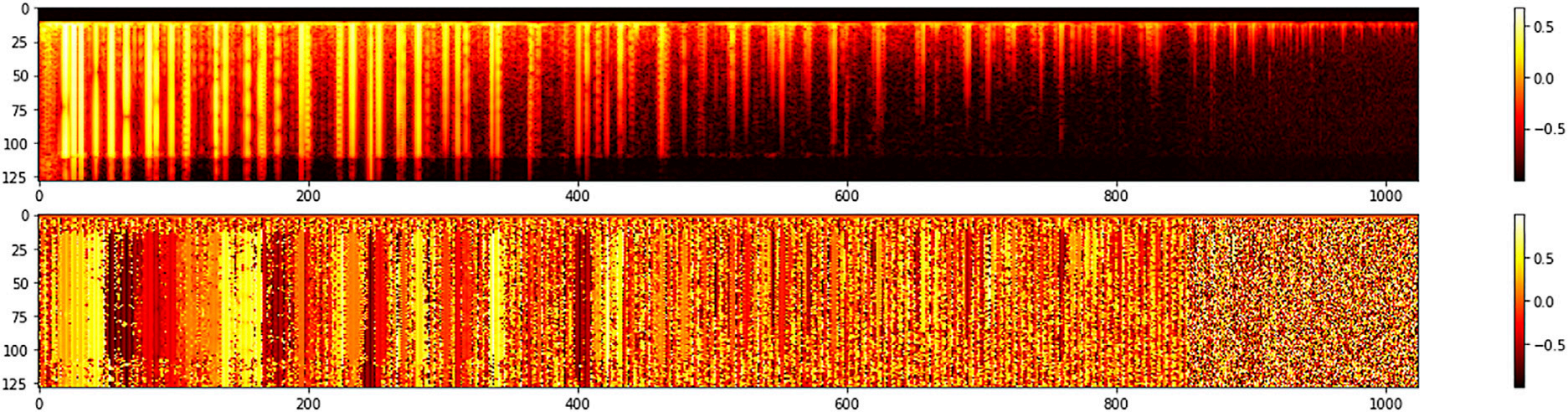
MFCC representation of a *forte* chord used for training. The horizontal dimension represents time while the vertical dimension encodes frequency coefficients. Brighter yellow colors represent higher sound intensities. The top graph shows the magnitude of the frequency representation and the bottom displays its phase.

**FIGURE 3:**
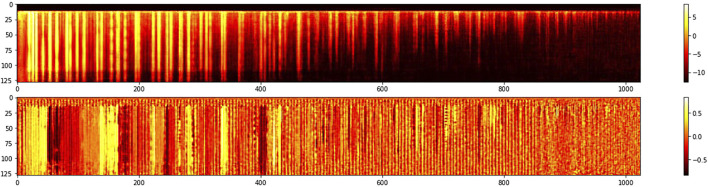
MFCC representation of the same forte chord of Figure 2 generated by the network’s decoder. The horizontal dimension represents time while the vertical dimension encodes frequency coefficients. Brighter yellow colors represent higher sound intensities. The top graph shows the magnitude of the frequency representation and the bottom displays its phase.

Figure 2 displays the MFCC transform of a 4-s audio recording of a piano chord performed *forte*. Figure 3 displays the MFCC representation of a 4-s audio recording of the same forte chord of Figure 2, but in this case, the chord was generated by the network by sampling a trained position in the latent space, the one where the original chord can be found. In both figures, 2 and 3, magnitude is shown on the top half while unwrapped phase is displayed at the bottom part.

We used Tensorflow 2.0 to implement our model. For training, we split our dataset leaving 38,880 examples for training and validation, and 4,320 examples for testing. We used an Adam optimizer with default parameters and learning rate of $3\times 10^{-5}$. We chose a batch size of 10, and the training was performed for a total of 250 epochs. The full training was done in about 6 h using one GPU, a Nvidia GTX 1080Ti. We used the standard cost function for VAE descried in Eq. 1, but in practice the model was trained to maximize the ELBO (Evidence Lower BOund) as proposed by Kingma and Welling (2014); Ranganath et al. (2014). We divided the 250 training epochs in five groups of 50 epochs. We started with a high reconstruction loss factor for the first 50 epochs and we decreased this factor across each epoch group. The high reconstruction loss factor allows for a good audio quality and then the later low reconstruction loss factor orders and clusters the latent space without a loss in audio quality (Higgins et al., 2017).

#### 4.1.2 Latent Space

**FIGURE 4:**
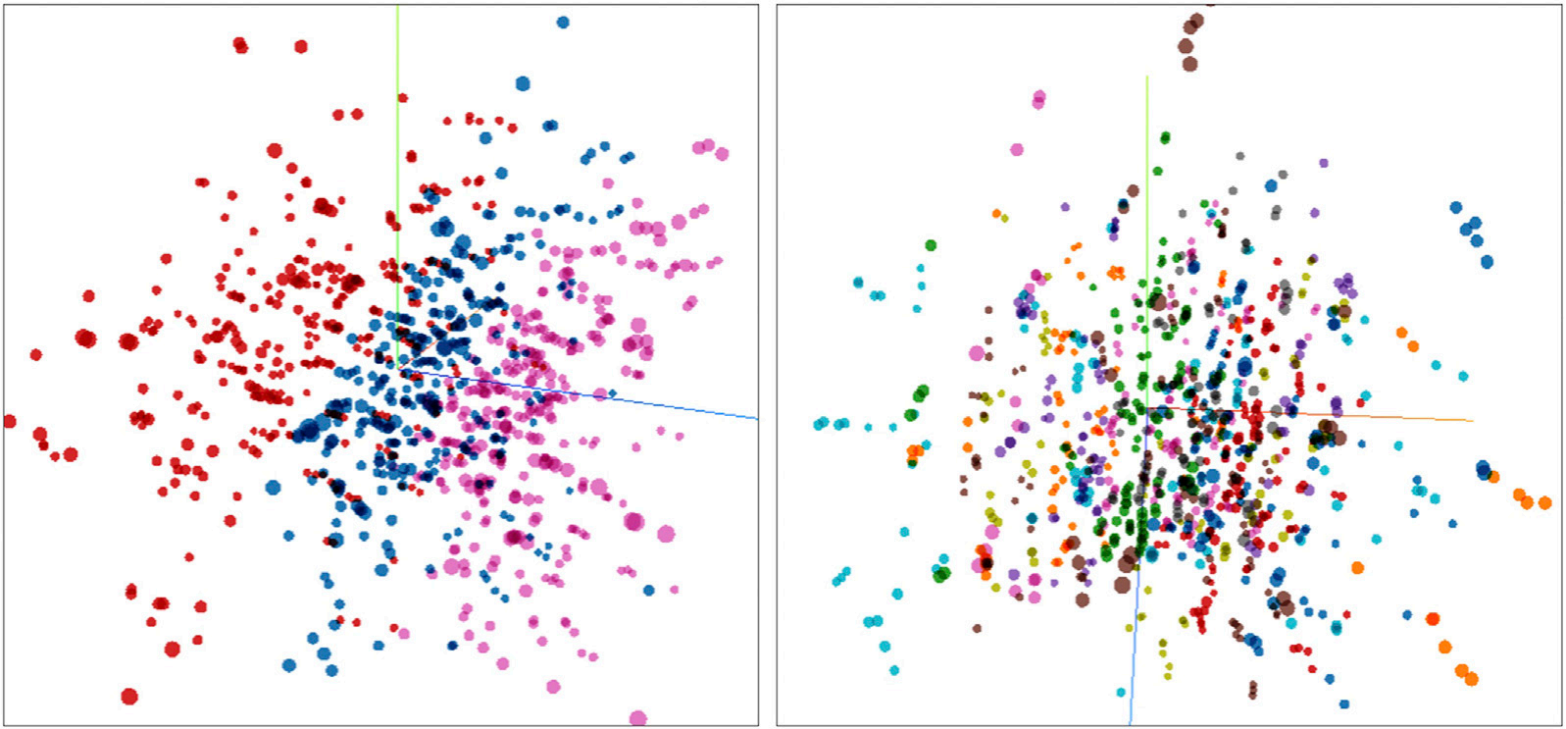
Three dimensional latent space representation of the input dataset. Left: Chords colored by volume (forte, mesoforte, and piano). Right: chords colored by base notes. It can be appreciated that the latent space is segmented based on relevant musical properties.

Figure 4 displays a three dimensional latent space generated by the network. On a macro level, chords are separated according to dynamic level as it can be observed on the right-most figure. On a micro level, chords are grouped with other chords with the same notes, and the nearest neighbors corresponds to the chords which have the most notes in common. This particular configuration of the latent space is very interesting from a musical stand point, as it appears that the networks learned to order the space based on musical concepts that are very fundamental such as common voicing, loudness and pitch.

**FIGURE 5:**
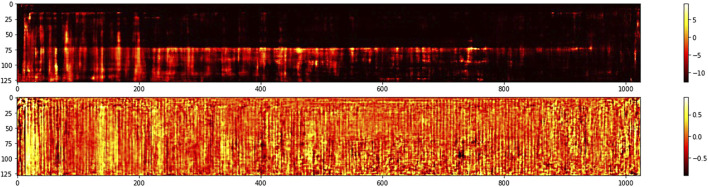
MFCC of a new chord generated by the network’s decoder by sampling an untrained point in the latent space. The horizontal dimension represents time while the vertical dimension encodes frequency coefficients. Brighter yellow colors represent higher sound intensities. The top graph shows the magnitude of the frequency representation and the bottom displays its phase.

**FIGURE 6:**
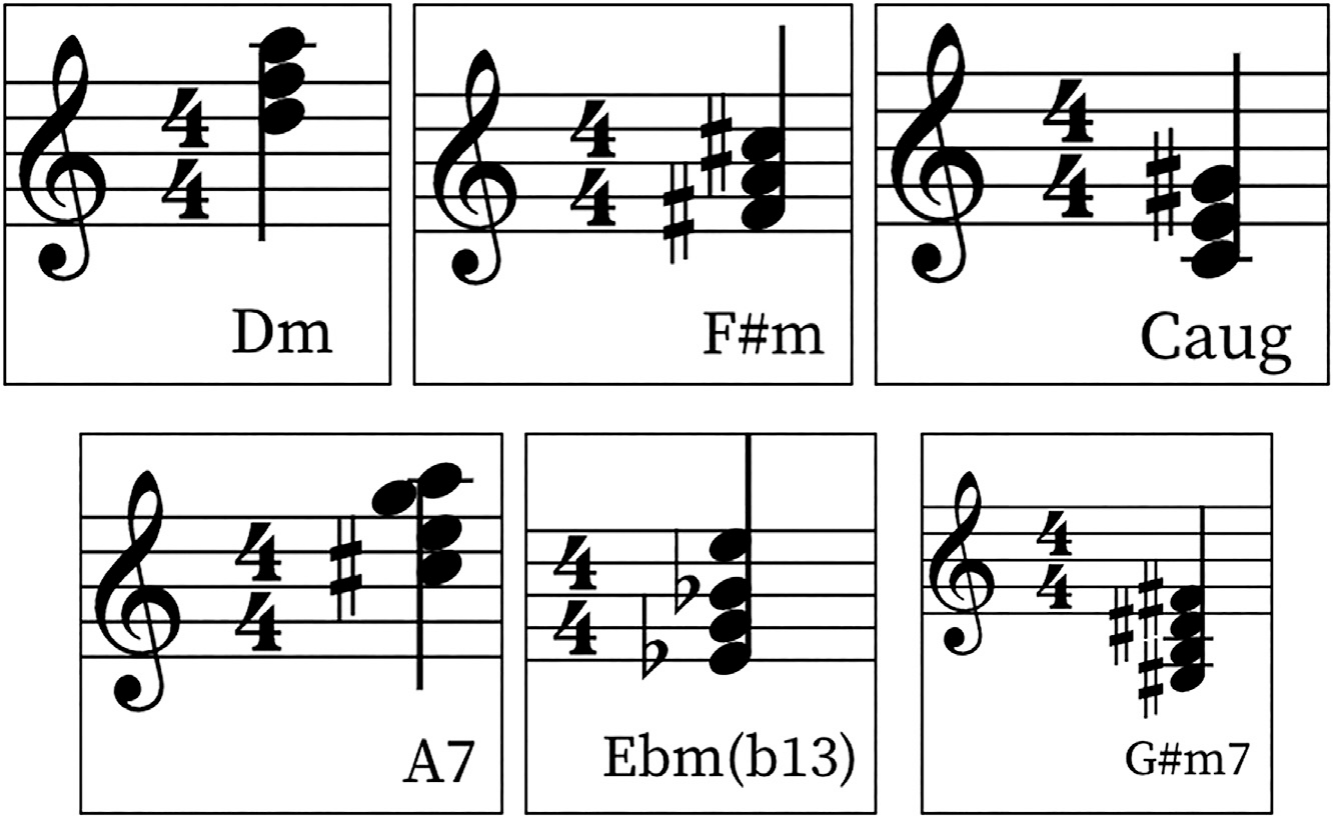
New chords generated by the TimbreNet model. The top three chords are new but they are similar to chords that can be found in the training set. The bottom three chords are completely novel, with different number of notes and representing different tonal functions, such as a dominant seventh or minor-minor seventh chords..

One of the nice properties of latent spaces happens when one samples the space in an untrained position, a point in the plane that has not been previously trained by the network. In Figure 5 we show the MFCC coefficients of a completely new chord generated by the network. Since different chords are clustered in the latent space, it is interesting to listen to chords that are generated in the space between clusters. We find out that the model is able to generate new chords with musical meaning that the model has never seen in the training dataset. Figure 6 shows some examples of new chords generated by the network. The top three chords can be found in the dataset while the bottom three chords are four note chords that the model has never seen before during training.

We have created an interactive web-based tool for the exploration of this latent space, called Timbreplay.^3^, in the same spirit of Moodplay (Andjelkovic et al., 2016, 2019) One nice feature of this tool is the generation of chord trajectories than the user can save for later use in musical compositions. In addition, audio examples of TimbreNet can be listened in the repository of the project.^4^.

### 4.2 StyleGAN Pianorolls: A Creative Musical Excerpts Generator

**FIGURE 7:**
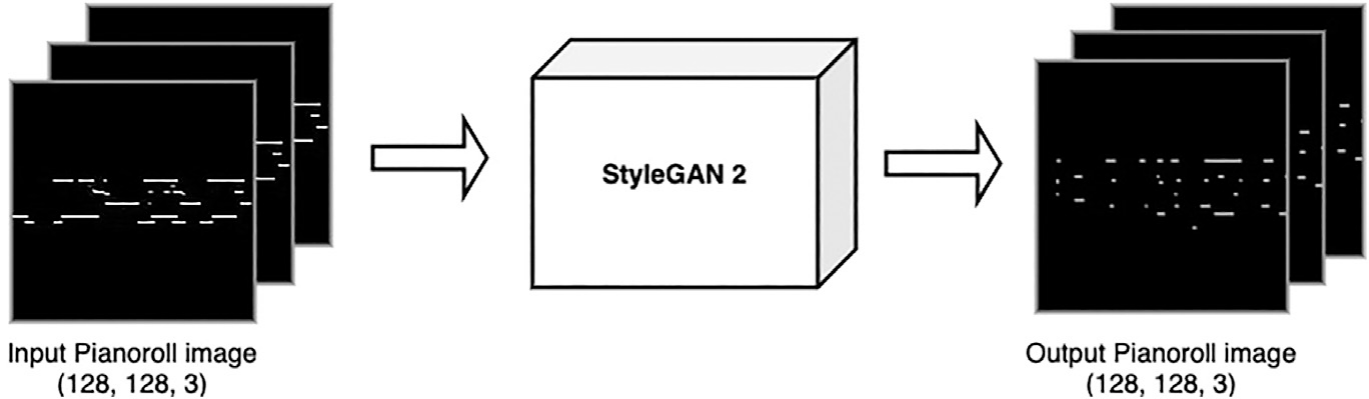
Representation of input and output of the StyleGAN 2 network with piano rolls. A symbolic representation of a musical excerpt in the form of a 128 × 128 × 3 piano roll is used to train the network. The output is another piano roll, which can later be transformed into midi or audio.

Our second case study is based on the newly developed StyleGAN 2 (Karras et al., 2020b), which achieved state-of-the-art results in image generation, specifically on creating human faces that do not exist, but look highly realistic. Considering this results for generating images, which are a 2D representation of visual information, we experimented to see if this network could generate piano rolls, which are also a 2D representation, but in this case of a musical composition, with one axis representing time, and the other representing pitches, as it can be seen in Figure 7 where both the input and output of the network are piano rolls represented as binary images. Even though we are fully aware that piano rolls are conceptually very different from human faces, we wanted to see if certain properties of visual information that they might have in common could be useful for training a musical generator model.

#### 4.2.1 Dataset and Model Training

We used the implementation of StyleGAN2-ADA in Tensorflow 1.14 provided by Karras et al. (2020a) that has an adaptive discriminator augmentation to better train with limited data. As inputs, we used the MAESTRO dataset V2.0.0 (Hawthorne et al., 2018), that consists of over 200 h of piano performances, which include raw audio and midi, 1,282 in total. We only used midi files, which can be very easily transformed to piano rolls. For each performance, its midi file was binarized and split into segments of 4 bars divided into 32 time steps each. After removing empty splits this processing resulted in 269,719 pianoroll images of shape (128, 128), this decision was made because the StyleGAN architecture has a constraint of using squared shape images. Although this constraint implies shorter musical segments, there’s still interesting information to be captured in the training data. We used the same loss function of Eq. 2. The model was trained on a Tesla V100 in Google Colab from a previously trained checkpoint on the FFHQ Dataset (Karras et al., 2018) which consists of human faces from Flickr. Surprisingly, even though the network previously knew human faces only, it was relatively easy to have it recognize and generate musical excerpts, as we detail below.

#### 4.2.2 Latent Space

One of the creative features of using the StyleGAN 2 architecture is that its random noise input is mapped into a disentangled latent space, called the *w*-space, through multiple fully connected layers. This new latent space is much richer to explore than the traditional latent space usually used in GANs, known as the *z*-space. The objective of using this disentangled *w*-space was to better separate different characteristics of the network’s output, allowing a much finer control of the generation process when producing new content.

For notated music this space has a lot of potential for further exploration. For example, one appealing idea is finding trajectories in the latent *w*-space that can change a specific characteristic of the output without changing other features, which means keeping other musical features constant. Some examples of desired musical changes can be the number of pitches in the excerpt, its tonal key, the amount of silences, or the amount of polyphony, among other interesting musical features that can be described in a piano roll representation.

**FIGURE 8:**
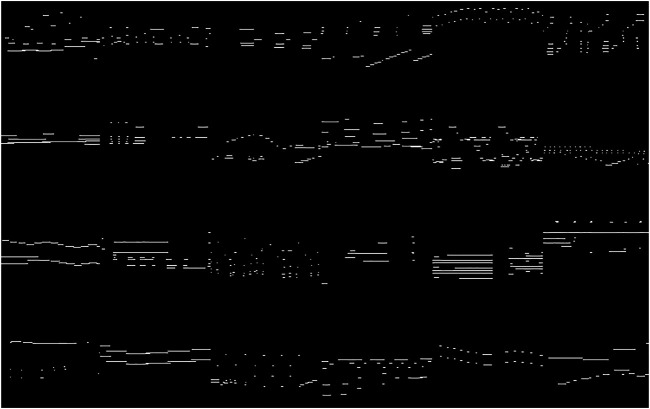
24 examples of real piano rolls used to train StyleGAN Pianorolls arranged in 4 rows and 6 columns. The examples exhibit great variation in their musical structure.

**FIGURE 9:**
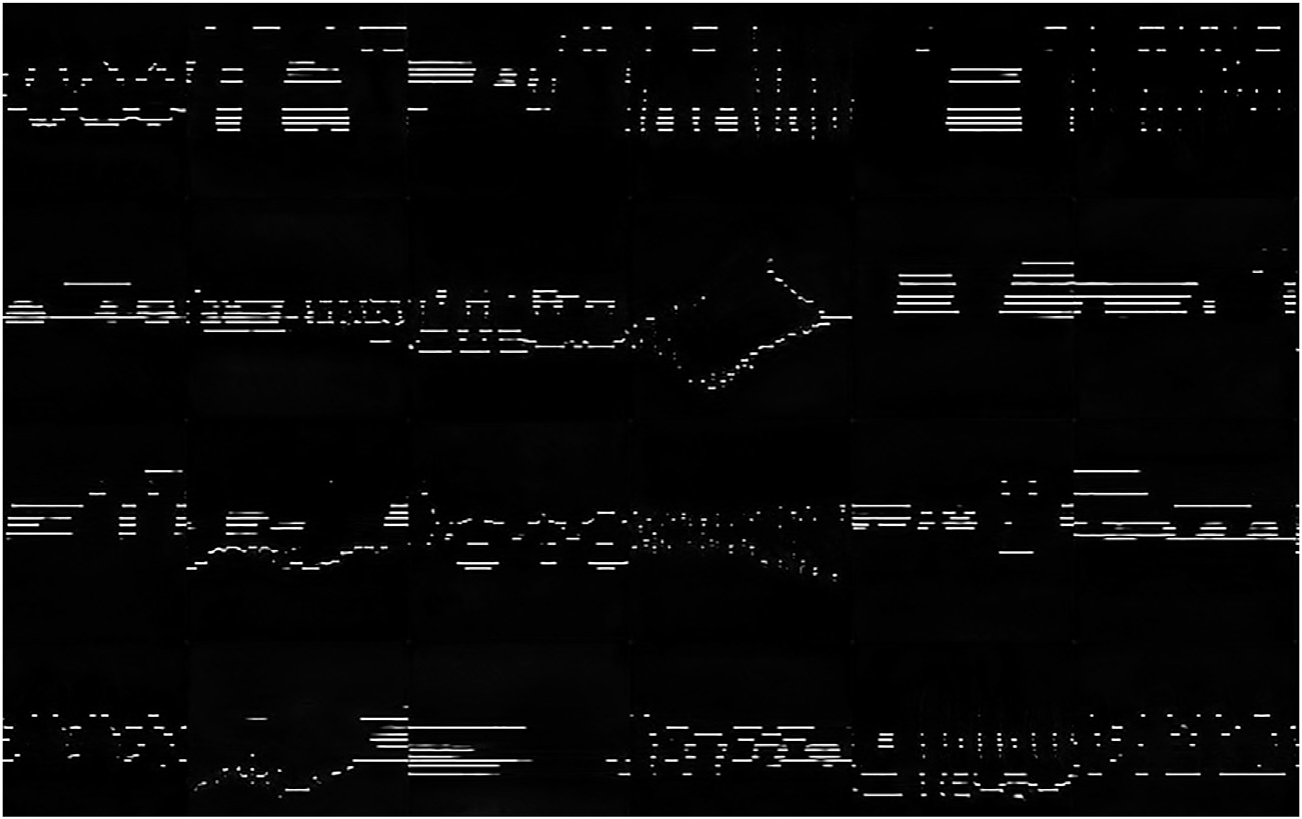
24 examples of fake piano rolls generated by StyleGAN Pianorolls arranged in 4 rows and 6 columns. The generated excerpts exhibit great variation in their musical structure, as it is the case of the input data.

**FIGURE 10:**
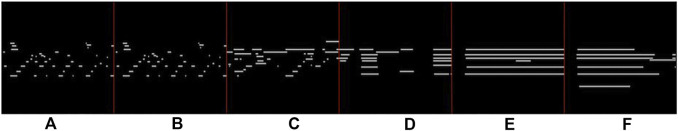
StyleGAN Pianorolls is able to generate a variety of musical ideas **(A–F)**. The latent space can also be interpolated between 2 outputs to generate a musically-meaningful sequence. In this case, the generated sequence exhibits how the network morphs from sample A to sample F in 4 steps, visually divided by a red line for easier differentiation.

In Figures 8, 9 there’s a comparison of several real input images against fake ones that were generated by our network. A first visual inspection of the images reveal that the fake images look very similar to the real ones. In terms of musical structures and motifs. the network is able to generate a great variety of musical ideas, ranging from pointillistic short events, as it can be observed in Figure 10A, to long chordal structures such as in Figure 10F. By interpolation of the latent space, it is also possible to generate a musical progression from one sample to another, with a variable number of intermediate steps, as Figure 10 depicts. For a more musical evaluation, we published a folder.^5^ with some selected samples to examine what this approach can potentially generate. There are single samples, which are the direct output of the network translated to MIDI and transformed to an audio file using Timidity++, and also sequence samples that are the concatenation of multiple outputs interpolated from two points in the *w*-space, further explanation of the types of generation will be explained in the next section.

#### 4.2.3 Generation

The process of generating an audio file from the output image of StyleGAN 2 has two parts: 1) defining a threshold and tempo for the generated piece, and 2) transforming the image to a numerical matrix. The first step is needed because the model returns a grayscale image, where the pixel values are between 0 and 255, and has three channels. With the threshold defined, we took the mean of the three channels and binarized the image using this threshold to determine which pixels correspond to played notes, thus, obtaining the numerical matrix which can be transformed to a pianoroll using the pypianoroll package developed by Dong et al. (2018b) to later convert it to a MIDI file. For listening to these files we used Timidity++ to convert them to a wave file.

We can generate new musical excerpts using this model through exploration of the latent *w*-space, changing the input values to get new pieces. Another interesting musical application is to interpolate between two examples generated by the network, defining the number of steps we can generate a sequence of concatenated outputs while moving from one point in the latent space to another, as shown in Figure 10. In the supplemented folder there’s examples of different sequences from two random sampled points in the latent space, showing how the trained model evolves one excerpt into another in a series of steps.

## 5 Perceptual Evaluation

We designed a very simple survey to obtain a first approximation to the perceptual validity of our results aimed towards determining whether our StyleGAN piano rolls network was able to generate musical excerpts that could be judged to be creative by human beings. Given the well-known ability of GANs to create realistic portraits, we created two videos.^6^ based on StyleGAN2 content. The visual content was generated by a StyleGAN 2 neural network trained with publicly available images of portraits of the Chilean National Art Museum (Museo Nacional de Bellas Artes), in the same fashion described in Karras et al. (2020b). The audio content was generated using two versions of the StyleGAN piano rolls model, one trained with different instruments from the LAKH MIDI Dataset (Raffel, 2016), and the other with the MAESTRO dataset (Hawthorne et al., 2018). We curated different musical excerpts from these networks to assemble the complete musical pieces. Our work consisted mainly in organizing the different fragments generated to create longer structures with multiple instrumentations, instead of focusing on a single instrument. It is important to clarify that the audiovisual content was completely generated by StyleGAN 2 networks, that none of those faces that appear in video exist in reality and neither do the musical structures that can be heard, they were completely created by a machine. Only the temporal organization of the music was done with human intervention. Finally, to achieve the final audiovisual results we used the Lucid Sonic Dreams.^7^ library, which uses a StyleGAN2 model to explore its latent space by synchronizing the transitions with a given audio, creating interesting movements to the rhythm of the music.

We asked participants to assess the creativity of each of the videos in terms of their audiovisual, visual only and audio only content, by selecting a number in a Likert scale from 1 to 5. 1 corresponded to the label “Disagree”, while five indicated “Agree”. 3 indicated no preference towards any side. For both videos, we evaluated the level of agreement/disagreement with the following statements:

1) The audiovisual content of the video is creative
2) The visual content of the video is creative
3) The audio content of the video is creative

**FIGURE 11:**
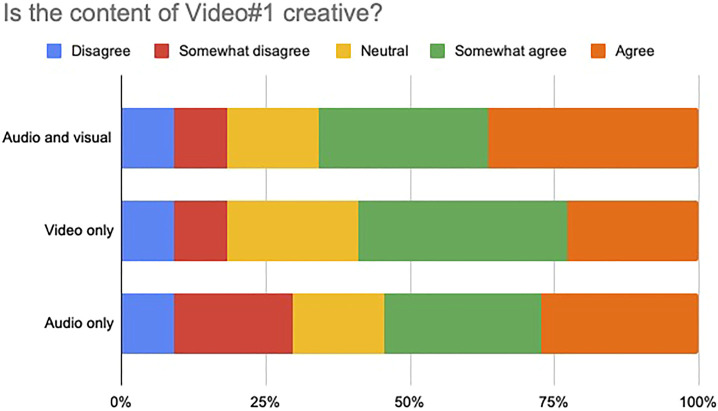
Perceptual evaluation of Video #1. Forty-four participants responded the survey over the internet. All three type of contents: audiovisual, visual only and audio only, were judged to be creative by a great majority of participants.

**FIGURE 12:**
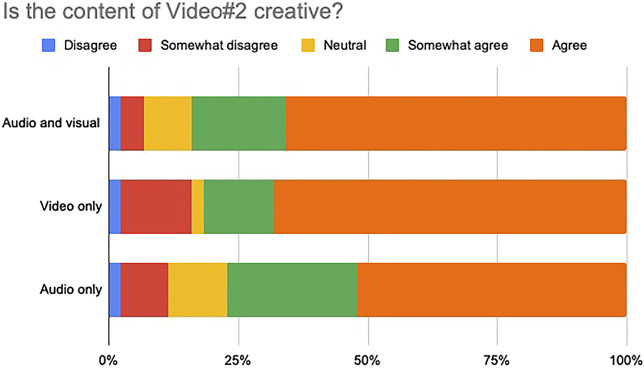
Perceptual evaluation of Video #2. Forty-four participants responded the survey over the internet. All three type of contents: audiovisual, visual only and audio only, were judged to be creative by a great majority of participants.

Forty-four participants responded the survey over the internet. The results are shown in Figures 11, 12, respectively. It is very clear, for both videos, that the majority of the subjects were in agreement with the statement that the content was creative. All three type of contents: audiovisual, visual only and audio only, especially in the second video, were judged to be creative by a great majority of participants.

## 6 Discussion

We believe that both of the case studies that we have presented exhibits certain aspects of computational creativity. In particular, both networks can generate novel musical excerpts or chords different from the ones contained in their respective training sets, a clear signal of novelty, but that also make musical sense and can function very well in their musical context at the same time, a probable sign of value. In TimbreNet, the network is trained with only tertian triad chords, consisting of only three notes arranged by thirds, and exclusively major, minor, diminished or augmented chords. However, as Figure 6 shows, the network can generate seventh chords, chords that are still tertian, but that contain four notes and that play a fundamental role in Western music, as their function within an harmonic context can be, for example, the dominant leading the way to the tonic, as in the case of the dominant seventh. If a chord-generating neural network trained with tertian triads exclusively can generate, after training, a dominant or minor-minor seventh chord, musical entities that the network had no clue they existed at all, does that make it a creative artificial intelligence? We believe that the answer must be yes, as the concept of a seventh chord is at the core of musical knowledge, and it is not trivial to derive from only regular tertian triads. In terms of value, it is interesting to notice that the network kept the configuration of new chords based on thirds, which makes musical sense. It could have simply generated lots of cluster chords, without any specific interval configuration, which would make them less coherent from a traditional Western harmonic point of view.

In general, a generative model is satisfactory if: 1) it can generate examples that appear to be drawn from the same distribution as the training dataset, a concept known as fidelity, and 2) the examples are suitably different from the examples shown during training, in other words, diversity (Naeem et al., 2020). In musical terms we can relate fidelity with adhesion to musical standards and diversity related to novelty and unexpectedness, all aspects of musical creativity (Daikoku et al., 2021). In the case of our experiments we found different degrees of achievement in fidelity and diversity depending on the number of dimensions of the latent space. For models with smaller latent space (3 or 4 dimensions) we found that the new chords were very similar to the chords in the dataset and no new different chords were generated, achieving fidelity but not diversity. For models with eight dimensions the chords were similar to the chords in the dataset but new chords with 4 or five notes were found. These new chords are suitably different from the training examples and they still have musical meaning and sense. We can say that this model achieved both fidelity and diversity. For models with bigger latent spaces (16 and 32 dimensions) new chords can be very different from those contained in the training set and they start loosing musical meaning and sense, achieving diversity, maximizing unexpectedness, but minimizing fidelity.

This network generates new chords when its latent space is sampled at coordinates that were not explicitly explored during training. It is indeed this sampling of uncharted territory that gives the possibility of something new and novel. This latent space is very similar to Boden’s idea of a structured conceptual space, and this process of exploration is very congruent with the concept of exploratory creativity (Boden, 2004). This idea is also supported by Franceschelli and Musolesi (2021) and Basalla and Schneider (2020), who claim that VAEs are the best possible computational examples of exploratory creativity, as their main goal is to create a structured compressed space open to further exploration.

How is it possible that the network learned the concept of a seventh chord? We don’t exactly know that at this point, but we propose that the fact that it learned that is a clear sign of creativity. One thing is being able to generate new audio based on chords, but a totally different thing is the ability to generate new chords, directly in audio, that fulfill a different tonal function with a different number of notes, but keeping its internal interval arrangement. In order to do that, TimbreNet must have learned the idea that a chord contains notes, that it can contain a variable number of them (even though it only saw tertian triads at training), and that these notes must be separated by major or minor thirds, in order to form a seventh chord. These, we insist, are not trivial concepts in music theory.

The fact that GANs possess a non-directly generated latent space, because the generator never sees real examples, implies that the sampling process in these kind of networks is from a conceptual space that could be indeed different from the original one, leading not only to exploratory creativity, but possibly also to transformational creativity (Franceschelli and Musolesi, 2021; Basalla and Schneider, 2020).

In effect, in StyleGAN Pianorolls, as it can be seen in Figure 9 and heard in the audio examples, a network originally trained on images of human faces learned how to generate musical excerpts. Representations and features from the spatial domain of images were somehow transformed into musical ideas, a clear conceptual leap, and an example of transformational creativity (Boden, 2004). These musical ideas are also novel and posses musical value, a fact that strengthens the creative aspects of this network.

This network also exhibits self-evaluation, another critical aspect of creativity according to Moruzzi (2021). The discriminator does not allow “false” examples to survive, relying only on true examples to improve its performance. In a sense, these networks know “when to stop”, without any need of external feedback.

The understanding of how the StyleGAN 2 model can learn to map different musical features, such as chords, scales and repeating motifs, to a new latent space to generate musical ideas that were not explicitly included in the training data, provides a new investigation opportunity to further explore how to use the disentangled space to get more control over the output of these models, so composers can use this as a tool for getting creative new ideas to overcome writers block for example.

In terms of the perceptual evaluation of the StyleGAN 2 model, we are aware that this is not a complete and rigorous perceptual evaluation of the creativity of our case studies. We also acknowledge that were are not comparing the results of these networks with those of a human counterpart, and that there is still some human intervention in the video production stage. Nevertheless, these results tend to confirm our hypothesis that these networks exhibits some traits of creativity, as their products were judged by a majority of our human subjects to be creative.

In summary, we have provided evidence that combined suggest that deep generative neural networks, can be, effectively, considered to be creative, or at least as creative as we consider humans are, based on our current understanding and knowledge on the topic. These networks generate valuable and novel outputs, and can conceptually leap, by using existing knowledge from a particular domain to generate knowledge in another domain. In the particular case of robot-generated music, these findings are particularly appealing and open a wide door for future creative possibilities.

## 7 Conclusion and Future Work

The spectacular development of DL has not been alien to the world of the arts, as recent advances in generative models have made possible the creation of deep creative networks. As an example, we presented two case studies of our own: TimbreNet, a VAE network trained to generate audio-based musical chords, and StyleGAN Pianorolls, a GAN capable of creating short musical excerpts. We discussed and assessed these generative models in terms of their creativity and we show that they are capable of learning musical concepts that are not obvious based on the training data, they exhibit novelty, diversity, self-assessment, they can also produce conceptual leaps, and exploratory and transformational creativity. We have shown that these deep models, based on our current understanding of creativity in robots and machines, can be considered, in fact, creative.

In particular, we focused on the aspects of 1) novelty, in the sense that these models should produce something that is not expected, 2) value, by assessing whether novel outputs function well in a musical context, 3) exploratory creativity as they can represent complex ideas in a compact conceptual space, 4) self-assessent, in the sense that they do know when to stop, and 5) diversity transformational creativity and conceptual leaps, where one type of knowledge is used to produce a different kind.

For the purposes of this article, we used an evaluation strategy based both on the first and second strategies proposed by Jordanous (2019): first a creative-practitioner-type approach, i.e., perceptual evaluations by humans, and second, based on the assessment of the authors, as we were the creators of both models. In future work, we would like to incorporate more evaluation strategies in order to strengthen the argument that these networks can exhibit creative behavior, and a more complete subjective evaluation by humans. And also, we would like to dive in more depth into the exploration of the latent spaces of both modes, not only to show that these networks can be creative, but to understand why: what have they learned and how they acquired that knowledge and why it is that they can consider creative.

## Data Availability

The datasets presented in this study can be found in online repositories. The names of the repository/repositories and accession numbers can be found below: https://zenodo.org/record/4740877 and https://zenodo.org/record/4747698.

## Footnotes

1 http://magenta.tensorflow.org.

2 https://github.com/CreativAI-UC/TimbreNet/tree/TimbreNet2/datasets/pianoTriadDataset.

3 http://timbreplay.ml.

4 https://github.com/CreativAI-UC/TimbreNet/tree/TimbreNet2/generated_chords/paper_examples.

5 https://drive.google.com/drive/folders/1cj-Y38GMxg4m0REWyU3ZvIUtWtp-TiOS?usp=sharing.

6 https://youtu.be/THxEOvRG4Ss, https://youtu.be/6CVAzQiMPlM.

7 https://github.com/mikaelalafriz/lucid-sonic-dreams.

## References

[B1] Andjelkovic I. Parra D. O'Donovan J. (2016). “Moodplay,” in UMAP 2016 - Proceedings of the 2016 Conference on User Modeling Adaptation and Personalization (Amsterdam, Netherlands: International Journal of Human-Computer Studies, Elsevier), 275–279. 10.1145/2930238.2930280

[B2] Andjelkovic I. Parra D. O’Donovan J. (2019). Moodplay: Interactive Music Recommendation Based on Artists' Mood Similarity. Int. J. Human-Computer Stud. 121, 142–159. 10.1016/j.ijhcs.2018.04.004

[B3] Basalla M. Schneider J. (2020). Creativity of Deep Learning: Conceptualization and Assessment, 1–12.

[B4] Boden M. A. (2009). Computer Models of Creativity. AIMag 30, 23–34. 10.1609/aimag.v30i3.2254

[B5] Boden M. A. (2004). The Creative Mind: Myths and Mechanisms. 2nd editio edn. London and New York: Routledge.

[B6] Bretan M. Weinberg G. (2016). A Survey of Robotic Musicianship. Commun. ACM 59, 100–109. 10.1145/2818994

[B7] Briot J.-P. Hadjeres G. Pachet F.-D. (2020). Deep Learning Techniques for Music Generation. Cham, Switzerland: Springer Nature. 10.1007/978-3-319-70163-9 Deep Learning Techniques for Music Generation.

[B8] Brown R. T. (1989). “Creativity. What Are We to Measure?,” in Handbook of Creativity. Perspectives on Individual Differences. Editors Glover J. A. Ronning R. R. Reynolds C. R. (Boston, Mass: Springer). 10.1007/978-1-4757-5356-1-1

[B9] Cádiz R. F. (2020). Creating Music with Fuzzy Logic. Front. Artif. Intell. 3, 1–20. 10.3389/frai.2020.00059 33733176 PMC7861252

[B10] Carnovalini F. Rodà A. (2020). Computational Creativity and Music Generation Systems: An Introduction to the State of the Art. Front. Artif. Intell. 3. 10.3389/frai.2020.00014 PMC7861321 33733133

[B11] Carter S. Nielsen M. (2017). Using Artificial Intelligence to Augment Human Intelligence. Distill 2, e9. 10.23915/distill.00009

[B12] Charniak E. (2018). Introduction to Deep Learning. MIT Press.

[B13] Collins D. (2007). Real-time Tracking of the Creative Music Composition Process. Digital Creativity 18 (4), 239–256. 10.1080/14626260701743234

[B14] Colton S. Halskov J. Ventura D. Gouldstone I. Cook M. Ferrer B. P. (2015). ICCC, 189–196.The Painting Fool Sees! New Projects with the Automated Painter.

[B15] Cope D. (1996). Experiments in Musical Intelligence. Madison, WI: A-R Editions.

[B16] [Dataset] Daikoku T. Wiggins G. A. Nagai Y. (2021). Statistical Properties of Musical Creativity: Roles of Hierarchy and Uncertainty in Statistical Learning. Front. Neurosci. 15. 10.3389/fnins.2021.640412 PMC8093513 33958983

[B17] Deng J. Kwok Y. K. (2016). “A Hybrid Gaussian-HMM-Deep-Learning Approach for Automatic Chord Estimation with Very Large Vocabulary, Editors Devaney, J. Mandel, M. Tzanetakis, G. Turnbull, D.,” in Proceedings of the 17th International Society for Music Information Retrieval Conference, ISMIR 2016, August 7-11, 2016, New York City, USA, 812–818.

[B18] Dong H.-W. Hsiao W.-Y. Yang L.-C. Yang Y.-H. (2018a). “Musegan: Multi-Track Sequential Generative Adversarial Networks for Symbolic Music Generation and Accompaniment,” in Thirty-Second AAAI Conference on Artificial Intelligence, New Orleans, Louisiana, New Orleans, LO.

[B19] Dong H.-W. Hsiao W.-Y. Yang Y.-H. (2018b). “Pypianoroll: Open Source Python Package for Handling Multitrack Pianorolls, Editors Flexer, A. Peeters, G. Urbano, J. Volk, A.,” in 19th International Society for Music Information Retrieval Conference (ISMIR), September 23-27, 2018, Paris, France, 1–2.

[B20] Edmonds E. Bilda Z. Muller L. (2009). Artist, Evaluator and Curator: Three Viewpoints on Interactive Art, Evaluation and Audience Experience. Digital Creativity 20, 141–151. 10.1080/14626260903083579

[B21] Eigenfeldt A. Burnett A. Pasquier P. (2012). “Evaluating Musical Metacreation in a Live Performance Context, Editors Lou Maher, M. Hammond, K. Pease, A. Pérez y Pérez, R. Ventura, D. Wiggins, G.,” in Proceedings of the 3rd International Conference on Computational Creativity, ICCC 2012, May 30 - June 1, 2012, Dublin, Ireland, 140–144.

[B22] Elgammal A. Liu B. Elhoseiny M. Mazzone M. (2017). CAN: Creative Adversarial Networks, Generating ”Art” by Learning about Styles and Deviating from Style Norms. ArXiv , 1–22.

[B23] Engel J. Hantrakul L. Gu C. Roberts A. (2020). ICLR, 1–19.DDSP: Differentiable Digital Signal Processing.

[B24] Engel J. Resnick C. Roberts A. Dieleman S. Eck D. Simonyan K. (2017). Neural Audio Synthesis of Musical Notes with Wavenet Autoencoders. arXiv preprint arXiv:1704.01279.

[B25] Franceschelli G. Musolesi M. (2021). Creativity and Machine Learning: A Survey.

[B26] Goodfellow I. Bengio Y. Courville A. (2016). Deep Learning. MIT Press.

[B27] Goodfellow I. Pouget-Abadie J. Mirza M. Xu B. Warde-Farley D. Ozair S. (2014). Advances in Neural Information Processing Systems, 2672–2680. Generative Adversarial Nets.

[B28] Grace K. Maher M. L. (2019). “Expectation-Based Models of Novelty for Evaluating Computational Creativity,” in Computational Creativity, Computational Synthesis and Creative Systems. Editors Veale T. Cardoso F. A. (Springer), 195–209. 10.1007/978-3-319-43610-4-9

[B29] Guzdial M. Riedl M. (2019). “Combinets: Creativity via Recombination of Neural Networks, Editors Grace, K. Cook, M. Ventura, D. Lou, M.,” in Proceedings of the 10th International Conference on Computational Creativity 2019, June 17-21, 2019, Charlotte, NC, 180–187.

[B30] Hadjeres G. Pachet F. Nielsen F. (2016). Deepbach: a Steerable Model for Bach Chorales Generation. arXiv preprint arXiv:1612.01010.

[B31] Hantrakul L. Kondak Z. Weinberg G. (2018). Practice Makes Perfect: Towards Learned Path Planning for Robotic Musicians Using Deep Reinforcement Learning. dl.acm.org 10.1145/3212721.3212839

[B32] Hawthorne C. Stasyuk A. Roberts A. Simon I. Huang C.-Z. A. Dieleman S. (2018). Enabling Factorized Piano Music Modeling and Generation with the MAESTRO Dataset, International Conference on Learning Representations, May 6-9, 2019. New Orleans, LO: ICLR.

[B33] Hennequin R. Khlif A. Voituret F. Moussallam M. (2019). Spleeter: a Fast and State-Of-The Art Music Source Separation Tool with Pre-trained Models. ISMIR.

[B34] Hertzmann A. (2018). Can Computers Create Art?. Arts 7, 18. 10.3390/arts7020018

[B35] Higgins I. Matthey L. Pal A. Burgess C. Glorot X. Botvinick M. (2017). “B-VAE: Learning Basic Visual Concepts with a Constrained Variational Framework,” in 5th International Conference on Learning Representations, ICLR 2017 - Conference Track Proceedings, April 24-26, 2017, Toulon, France, 1–22.

[B36] Humphrey E. J. Cho T. Bello J. P. (2012). “Learning a Robust Tonnetz-Space Transform for Automatic Chord Recognition,” in ICASSP, IEEE International Conference on Acoustics, Speech and Signal Processing - Proceedings, March 25-30, 2012, Kyoto, Japan, 453–456. 10.1109/ICASSP.2012.6287914

[B37] Jordanous A. (2019). “Evaluating Evaluation: Assessing Progress and Practices in Computational Creativity Research,” in *Computational Creativity,* Computational *Synthesis And Creative Systems*. Editors Veale T. Cardoso F. A. (Springer), 211–236. 10.1007/978-3-319-43610-4-10

[B38] Kalin J. (2018). Generative Adversarial Networks Cookbook. Birmingham, UK: Packt Publishing.

[B39] Karimi P. Grace K. Maher M. L. Davis N. (2018). Evaluating Creativity in Computational Co-creative Systems. arXiv preprint arXiv:1807.09886

[B40] Karras T. Aittala M. Hellsten J. Laine S. Lehtinen J. Aila T. (2020a). Training Generative Adversarial Networks with Limited Data

[B41] Karras T. Laine S. Aila T. (2019). A Style-Based Generator Architecture for Generative Adversarial Networksin Proceedings of the IEEE/CVF Conference on Computer Vision and Pattern Recognition, June 14-19, 2020, 4401–4410. Tech. Rep. 10.1109/TPAMI.2020.2970919 32012000

[B42] Karras T. Laine S. Aittala M. Hellsten J. Lehtinen J. Aila T. (2020b). “Analyzing and Improving the Image Quality of StyleGAN,” in Proceedings of the CVPR.

[B43] Kim H. Garrido P. Tewari A. Xu W. Thies J. Niessner M. (2018). Deep Video Portraits. ACM Trans. Graphics, 37 (4), 1–14. 10.1145/3197517.3201283

[B44] Kingma D. P. Welling M. (2014). “Auto-encoding Variational Bayes,” in 2nd International Conference on Learning Representations, ICLR 2014 - Conference Track Proceedings, April 14-16 2014, Banff, Canada, 1–14.

[B45] Korzeniowski F. Widmer G. (2016). “Feature Learning for Chord Recognition: The Deep Chroma Extractor, Editors Devaney, J. Mandel, M. I. Turnbull, D. Tzanetakis, G. ,” in Proceedings of the 17th International Society for Music Information Retrieval Conference, ISMIR 2016, August 7-11, 2016, New York City, USA, 37–43.

[B46] Krizhevsky A. Sutskever I. Hinton G. E. (2012). ImageNet Classification with Deep Convolutional Neural Networks. Adv. Neural Inf. Process. Syst. 10.1061/(ASCE)GT.1943-5606.0001284

[B47] Miranda E. R. Witkowski M. (2005). “Musical Composition by Autonomous Robots: A Case Study with AIBO, Editors Nehmzow, U. Melhuish, C. Tikhanoff, V.,” in Proceedings of Toward Autonomous Robotic Systems (TAROS), September 12-14 2005, London, UK.

[B48] Moruzzi C. (2018). “Creative AI: Music Composition Programs as an Extension of the Composer’s Mind,”. Studies in Applied Philosophy, Epistemology and Rational Ethics. Editor Muller V. C. (Springer), 44, 69–72. 10.1007/978-3-319-96448-5-8

[B49] Moruzzi C. (2021). Measuring Creativity: an Account of Natural and Artificial Creativity. Eur. J. Philos. Sci. 11, 1–20. 10.1007/s13194-020-00313-w

[B50] Mumford M. Ventura D. (2015). “The Man behind the Curtain: Overcoming Skepticism about Creative Computing, Editors Toivonen, H. Colton, S. Cook, M. Ventura, D.,” in Proceedings of the Sixth International Conference on Computational Creativity, June 29–July 2, 2015, Park City, Utah.

[B51] Naeem M. F. Oh S. J. Uh Y. Choi Y. Yoo J. (2020). Reliable Fidelity and Diversity Metrics for Generative Models. International Conference on Machine Learning. Vienna, Austria.

[B52] Oord A. v. d. Dieleman S. Zen H. Simonyan K. Vinyals O. Graves A. (2016). Wavenet: A Generative Model for Raw Audio. arXiv preprint arXiv:1609.03499.

[B53] Park Y. (2019). Can Artworks by Artificial Intelligence Be Artworks?. AM. J. Art Media Stud. 113. 10.25038/am.v0i20.332

[B54] Raffel C. (2016). Learning-Based Methods for Comparing Sequences, with Applications to Audio-To-MIDI Alignment and Matching. Ph.D. thesis. Columbia University.

[B55] Ranganath R. Gerrish S. Blei D. M. (2014). Black Box Variational Inference. J. Machine Learn. Res. 33, 814–822.

[B56] Ritchie G. (2019). “The Evaluation of Creative Systems,” in Computational Creativity, Computational Synthesis and Creative Systems,. Editors Veale T. Cardoso F. A. (Springer), 159–194. 10.1007/978-3-319-43610-4-8

[B57] Roberts A. Engel J. Eck D. (2017). Hierarchical Variational Autoencoders for Music. NIPS Workshop on Machine Learning for Creativity and Design, 3.

[B58] Roberts A. Engel J. Raffel C. Hawthorne C. Eck D. (2018). A Hierarchical Latent Vector Model for Learning Long-Term Structure in Music. arXiv preprint arXiv:1803.05428.

[B59] Rowe R. (2004). Machine Musicianship. Cambridge, MA: The MIT Press. 10.7551/mitpress/4361.003.0002

[B60] Sawyer R. K. (2006). Explaining Creativity. The Science of Human Innovation. Oxford University Press.

[B61] Sternberg R. J. Sternberg K. (2012). Cognitive Psychology. sixth edit edn. Belmont, CA: Wadsworth Cengage Learning.

[B62] Sturm B. L. Ben-Tal O. Monaghan U. Collins N. Herremans D. Chew E. (2018). Machine Learning Research that Matters for Music Creation: A Case Study. J. New Music Res. 48, 36–55. 10.1080/09298215.2018.1515233

[B63] Sturm B. L. Santos J. F. Ben-Tal O. Korshunova I. (2016). Music Transcription Modelling and Composition Using Deep Learning. *arXiv preprint arXiv:1604.*08723.

[B64] Weber A. Alegre L. N. Tørresen J. da Silva B. C. (2019). Parameterized Melody Generation with Autoencoders and Temporally-Consistent Noise, Proceedings of the New Interfaces for Musical Expression Conference, June 3-6, 2019. Porto Alegre, Brazil: NIME, 174–179.

[B65] Wyse L. (2019). “Mechanisms of Artistic Creativity in Deep Learning Neural Networks, Editors Grace, K. Cook, M. Ventura, D. Lou, M.,” in Proceedings of the 10th International Conference on Computational Creativity 2019, June 17-21, 2019, Charlotte, NC.

[B66] Yamshchikov I. P. Tikhonov A. (2020). Music Generation with Variational Recurrent Autoencoder Supported by History. SN Appl. Sci. 2, 1–7. 10.1007/s42452-020-03715-w

[B67] Yang L.-C. Chou S.-Y. Yang Y.-H. (2017). MidiNet: A Convolutional Generative Adversarial Network for Symbolic-Domain Music Generation. arXiv preprint arXiv:1703.10847.

[B68] Yang L. C. Lerch A. (2020). On the Evaluation of Generative Models in Music. Neural Comput. Appl. 32, 4773–4784. 10.1007/s00521-018-3849-7

[B69] Zhou X. Lerch A. (2015). “Chord Detection Using Deep Learning, Editors Müller, M. Wiering, F.,” in Proceedings of the 16th International Society for Music Information Retrieval Conference, ISMIR 2015, October 26-30, 2015, Málaga, Spain, 52–58.

